# *SPL36* Encodes a Receptor-like Protein Kinase that Regulates Programmed Cell Death and Defense Responses in Rice

**DOI:** 10.1186/s12284-021-00475-y

**Published:** 2021-04-07

**Authors:** R. A. O. Yuchun, J. I. A. O. Ran, W. A. N. G. Sheng, W. U. Xianmei, Y. E. Hanfei, P. A. N. Chenyang, L. I. Sanfeng, Xin Dedong, Z. H. O. U. Weiyong, D. A. I. Gaoxing, H. U. Juan, R. E. N. Deyong, W. A. N. G. Yuexing

**Affiliations:** 1 Zhejiang Provincial Key Laboratory of Biotechnology on Specialty Economic Plants, Zhejiang Normal University, Jinhua, 321004 China; 2 State Key Laboratory of Rice Biology, China National Rice Research Institute, Hangzhou, 310006 China; 3 Guangxi Academy of Agricultural Sciences, Nanning, 530000 China

**Keywords:** Defense response, Receptor-like protein kinase, Lesion mimic mutant, Rice, Salt resistance, *SPL36*

## Abstract

Lesion mimic mutants spontaneously produce disease spots in the absence of biotic or abiotic stresses. Analyzing lesion mimic mutants’ sheds light on the mechanisms underlying programmed cell death and defense-related responses in plants. Here, we isolated and characterized the rice (*Oryza sativa*) *spotted leaf 36* (*spl36*) mutant, which was identified from an ethyl methanesulfonate-mutagenized *japonica* cultivar Yundao population. *spl36* displayed spontaneous cell death and enhanced resistance to rice bacterial pathogens. Gene expression analysis suggested that *spl36* functions in the disease response by upregulating the expression of defense-related genes. Physiological and biochemical experiments indicated that more cell death occurred in *spl36* than the wild type and that plant growth and development were affected in this mutant. We isolated *SPL36* by map-based cloning. A single base substitution was detected in *spl36*, which results in a cysteine-to-arginine substitution in *SPL36*. *SPL36* is predicted to encode a receptor-like protein kinase containing leucine-rich domains that may be involved in stress responses in rice. *spl36* was more sensitive to salt stress than the wild type, suggesting that *SPL36* also negatively regulates the salt-stress response. These findings suggest that *SPL36* regulates the disease resistance response in rice by affecting the expression of defense- and stress-related genes.

**Supplementary Information:**

The online version contains supplementary material available at 10.1186/s12284-021-00475-y.

## Background

The lesion mimic phenotype is characterized by the spontaneous production of disease spots of various sizes and shapes on the leaves and leaf sheaths (and even stalks and seeds) in the absence of abiotic or biotic stress. Lesion mimics are the result of apoptosis caused by the hypersensitive response (HR) (Petrov et al., 2015). Lesion mimic mutants in rice can be divided into the initial (local) type and the spreading type based on phenotype and whether a dominant or recessive mutation is present. The first lesion mimic mutant in plants was reported in maize by the American scientist R. A. Emerson in the 1920s (Lu et al., 2012). *Sekiguchi Lesion* (*sl*), the first lesion mimic mutant identified in rice, was discovered by the Japanese scientist Sekiguchi in the mid-1960s as a naturally occurring mutant (Liu et al., 2004).

The mechanism underlying the generation of lesion mimics is complex and regulated by multiple factors, including both internal and external factors. Internal factors include the altered expression of disease resistance-related genes, uncontrolled programmed cell death (PCD), metabolic disorders, defense signaling molecules, and the loss of protease function; external factors include temperature and light. For example, *SPL7*, the first lesion mimic mutant gene successfully cloned in rice, encodes the heat shock protein HSFA4, a transcription factor that plays a negative role in the apoptosis pathway (Yamanouchi et al., 2002). *SPL7* is highly similar to maize *HSFb*, tomato *HSF8*, and Arabidopsis *HSF21* and *HSF1*, all of which regulate apoptosis in plants; mutants of these genes show lesion mimic characteristics. The phenotype of *spl18* mutant of rice is associated with the insertion of a T-DNA activation tag that enhances the expression of genes around the insertion site (Mori et al., 2007). *OsATL*, encoding an acyltransferase homolog that induces allergic reactions to tobacco, is located ~ 500 bp downstream of the inserted T-DNA activation tag. This gene is expressed at low levels in wild-type rice but at high levels in *spl18*, resulting in the occurrence of lesion mimics due to the abnormal expression of disease resistance genes.

Rice plants with mutations in *NLS1*, encoding a CC-NB-LRR protein, accumulate large amounts of H_*2*_O_2_ and salicylic acid and show abnormal expression of resistance-related genes, leading to the appearance of lesion mimics in leaf sheaths (Tang et al., 2011). The protein that is altered in the lesion mimic mutant *spl11* contains U-box and ARM (armadillo) repeat domains and undergoes ubiquitination and protein-protein interactions when expressed in yeast and mammalian systems (Zeng et al., 2002). The similarity of this protein to other plant U-box-ARM proteins is mainly limited to the U-box and ARM repeat regions. A single base substitution was detected in the mutant *spl11* gene, resulting in the premature termination of translation of the encoded proteins. The E3 ubiquitin ligase activity of this protein is dependent on the presence of an intact U-box domain, indicating that ubiquitination plays a role in plant cell death and defense and suggesting that spontaneously formed lesion mimics are associated with uncontrolled PCD (Zeng et al., 2004). *OsSSI2* encodes fatty acid dehydrogenase (FAD), which also plays a negative role in the defense response in rice. The loss of function of FAD results in lesion mimics and delayed leaf growth (Jiang et al., 2009). Mutations in a gene encoding uridine diphosphate-N-acetylglucosamine pyrophosphorylase (UAP1), which functions during glucose metabolism, can also lead to the appearance of lesion mimics in rice leaves (Jung et al., 2005).

Most lesion mimic mutants in rice show enhanced disease resistance to some extent. Among the more than 80 mutants that have been identified, 11 mutants (including *spl1*, *spl9*, *spl10*, *cdr1*, and *cdr3*) show enhanced blast resistance (Liu et al., 2004; Yoshimura et al., 1997; Takahashi et al., 1999); 12 mutants (including *spl21*, *spl24*, *lmes1*, *hm197*, and *hm83*) show enhanced bacterial blight resistance (Wu et al., 2008); 19 mutants (including *spl14*, *bl3*, and *Lmr*) show enhanced blast resistance and bacterial blight resistance (Mizobuchi et al., 2002); and one, *lmm1*, shows both enhanced blast resistance and sheath blight resistance. By contrast, the disease resistance of *spl2*, *spl3*, *spl4*, *spl6*, *spl7*, and *ncr1* is unchanged or even reduced compared to the wild type (Kang et al., 2007; Campbell and Ronald, 2005).

Plant receptor-like protein kinases (RLKs) occupy important metabolic positions and are abundant in plants; rice has approximately 1130 RLK genes (Nguyen et al., 2015). Plant RLKs are composed of intracellular, extracellular, and transmembrane regions (Ye et al., 2017). Most RLKs contain an extracellular receptor domain (ECLB), a transmembrane domain (TM), and a protein kinase contact response domain (PKC) (Walker, 1994; Zhang, 1998).

The leucine-rich repeat (LRR) receptor-like protein kinases are a subtype of RLKs that are involved in plant stress responses and defense-related processes, including disease responses. The Cf gene family of tomato leaf mold encodes proteins with LRR structures; differences in the amino acid sequences of the LRR motifs of different proteins in the same family are responsible for the specificity of ligand binding (Thomas et al., 1998). The resistance gene *FLS2* in Arabidopsis has a similar structure to the sequence encoding the extracellular domain of the tomato Cf gene family (Gómez-Gómez et al., 2001). Upon binding to ligands (avirulence gene products of rice bacterial blight pathogens), the extracellular LRR structure of rice *Xa21* induces intracellular kinase phosphorylation and produces a series of cellular responses that protect the plant from pathogens (Song et al., 1995; Park and Ronald, 2012). These findings indicate that the LRR structure plays an important role in identifying the basic structures of pathogens and microorganisms.

Here, to further explore the signal transduction pathways of LRR-type receptor kinases in response to stress signals in rice, we isolated and characterized the novel lesion mimic mutant *spotted leaf 36* (*spl36*). This mutant shows spots at the tillering stage and enhanced resistance to bacterial blight. We cloned the *SPL36* gene by map-based cloning and demonstrated that it encodes a receptor-like protein kinase receptor that is expressed in all tissues and at all developmental stages after the tillering stage and is localized to the plasma membrane. A high frequency of cell death, changes in chloroplast structure, and activation of defense-related responses were observed in the *spl36* mutant. We demonstrate that the loss of function of *SPL36* is responsible for the cell death, premature senescence, and activation of the defense response in this mutant.

## Materials and Methods

### Plant Materials and Growth Conditions

The spotted leaf mutant *spl36* was isolated from a methanesulfonate (EMS)-induced mutant library of Yundao rice (wild type, WT). The mutant was hybridized with TN1 as the male parent, and the F_1_ offspring and F_2_ population were grown in the rice experimental field of Zhejiang Normal University, Jinhua City, Zhejiang Province, China during the summer of 2018 and 2019. The F2 populations of both *spl36*/ZF802 and *spl36*/TN1 were used for genetic analysis, and the F2 recessive individuals of *spl36*/TN1 were used for gene fine mapping.The agronomic traits of wild-type and *spl36* plants were statistically analyzed, including plant height, tiller number, grain number per panicle, seed setting rate, and 1000-grain weight. The results were analyzed based on the average of 10 replicates.

### Measuring Photosynthetic Parameters and Chlorophyll Content

From 9:30 a.m.to 11:00 a.m. on sunny days, 10 individual plants with relatively uniform growth were harvested. The photosynthetic parameters of wild type and mutant plants were measured with an LI-6400XT portable photosynthesis tester. Three to five representative flag leaves were treated and measured, and each leaf was measured in triplicate (the mean value was taken as one replicate). During the measurement, red and blue light sources were used, the light intensity was constant at 1200 μmol/m^2^, the temperature was 30 °C, the CO_2_ concentration was the concentration in the air, and the humidity level was the relative humidity in the atmosphere. For chlorophyll measurements, five wild type and mutant plants with relatively uniform growth vigor were selected. The leaves were weighed, and 0.05 g of leaf tissue was cut into pieces and soaked in 25 mL 1:1 ethanol: acetone solution; three replicates were subjected to the darkening reaction for 24 h, followed by shaking. The absorbance values at 663 nm, 645 nm, and 470 nm were measured with a spectrophotometer, and the photosynthetic pigment content was calculated and statistically analyzed by Student’s t-test.

### Preparation of Rice Protoplasts

To generate rice protoplasts, 60 rice seedlings were cultured for 12–15 days. The leaves were removed from the plants with sterile scissors, leaving only the stalk. The material was cut into 0.5-mm strips and placed into a sterilized 50 mL triangular flask. After adding 30 mL 0.6 mol/L mannitol, the sample was incubated for 15 min with shaking in the dark at 28 °C and 50 r/min. After shaking, the mannitol solution was poured off, and the sample was transferred to a clean sterile triangular flask. Enzymatic hydrolysis was performed by adding 20 mL enzyme solution (1.5% fibrinase R-10, 0.75% segregation enzyme R-10, 0.6 mol/L mannitol, 10 mmol/L MES, 10 mmol/L CaCl_2_, 0.1% BSA, pH 5.7) and incubating at 28 °C with shaking at 60 ~ 80 R /min for 4 ~ 5 h. Following enzymatic hydrolysis, the same volume of W5 solution (154 mmol/L NaCl, 125 mmol/L CaCl_2_, 5 mmol/L KCl, 2 mmol/L MES, pH 5.7) was added to the sample. Pre-cooled. After 15 s of severe shock, the sample was passed through a nylon filter. The protoplasts were cleaned with W5 solution, centrifuged at 1000 r/min for 5 min, and the supernatant discarded; this step was repeated 3 to 5 times. Following the addition of MMg solution, the protoplasts were collected and examined by microscopy and incubated on ice for 40 min.

### Histochemical Analysis

The content and concentration of malondialdehyde (MDA) and the enzymatic activity of superoxide dismutase of peroxidase (POD) were analyzed following the manufacturer’s instructions (Nanjing Jiancheng Bioengineering Institute, Nanjing, China). The MDA and H_2_O_2_ contents and SOD and POD activity were measured when the tillering stage was first visible in *spl36*. Apoptosis was detected by TUNEL assay. FAA fixative was prepared before sampling and placed into a 2 mL centrifuge tube (Liang and Zhou., 2018). When the mutant lesion phenotype was apparent in *spl36* leaves, leaf tissues showing this phenotype were harvested, along with wild-type leaves at the corresponding position, cut into clumps, and placed in the 2/3 position of FAA fixative for fixation. The leaf tissue was vacuum infiltrated until it sank to the bottom. The tube was sealed with Parafilm and stored in a refrigerator at 4 °C. A TUNEL apoptosis detection kit (Roche, Cat No.1684817) was used to measure apoptosis in the samples (Inada et al., 1998).

### Linkage Analysis and Mapping of *spl36*

SSR primers that are evenly distributed over the 12 rice chromosomes (from our laboratory) were used to screen the mutants and TN1 for polymorphisms (Supplementary Table 2). Twenty-one individual F_2_ lesion mimic *spl36*/TN1 plants were used for linkage analysis to preliminarily confirm the chromosomal location of the target gene. A new InDel marker with relatively good polymorphism was developed in the mapping interval, and the target gene was precisely mapped using a single plant showing the mutant phenotype in the F_2_ segregating population of *spl36*/TN1. Genomic DNA was extracted from the samples using the hexadecyltrimethylammonium bromide (CTAB) method (Wu et al., 2008). The PCR mixture included 1 μL DNA template, 1 μL 10 × PCR buffer, 0.5 μL each of forward and reverse primers (10 μmol/L), 1 μL dNTPs, 0.2 μL rTaq, and H_2_O to a final volume of 10 μL. The PCR amplification program was as follows: pre-denaturation at 94 °C for 4 min; denaturation at 94 °C for 30 s, annealing at 55–60 °C for 30 s (depending on the primers), extension at 72 °C for 30 s, 40 cycles; and a final extension at 72 °C for 10 min. The PCR products were separated by electrophoresis on a 4% agarose gel, photographed, and the data stored in a gel imager and read. The primers used for mapping are shown in Supplementary Table 3.

### Vector Construction

For functional complementation of the *spl36* mutants, the complete genomic DNA fragment (including the promoter) of wild-type *SPL36* was amplified by PCR with primers *spl36*-CPT-F/ *spl36*-CPT-R and used to construct the vector for transformation by insertion into empty binary vector pCAMBIA1300 via Clontech In-Fusion PCR (TaKaRa). The full-length *SPL36* open reading frame (ORF) was amplified with the primer pair *spl36*-GFP-F/*spl36*-GFP-R, and the coding sequence of *SPL36* was inserted into binary vector pHQSN containing the 35S promoter (p*35S*::*SPL36*) for subcellular localization analysis. The *SPL36* promoter was introduced into the expression vector pCAMBIA1305.1, and the expression of *SPL36* in rice tissues was revealed using the GUS reporter gene. GFP fluorescence was observed by confocal laser-scanning microscopy (Leica TCS SP5, Leica, Germany). The primers used for vector construction are shown in Supplementary Table 4.

### Inoculation Test

*Xanthomonas oryzae* pv. *Oryzae, Xoo* (the causal agent of bacterial blight) was inoculated onto the flag leaves of wild-type Yundao and the *spl36* mutant at the tillering stage using the leaf clipping method (The selected leaves should be fresh and free of disease spots and senescence). Specifically, healthy, fully unfolded rice leaves were selected, and the tip of each leaf (~ 1 cm) was cut with scissors that were dipped into *Xanthomonas oryzae* pv. *Oryzae, Xoo* solution before making each cut. The phenotypes of the inoculated leaves were observed at 5 and 10 days after inoculation and the lesion length measured and photographed.

### Quantitative Reverse-Transcription PCR Analysis

Leaf, root, stem, leaf sheath, panicle, and grain samples were collected from wild type and mutant plants at each stage of development. RNA was extracted from the samples using an RNAprep Pure Plant Kit (Cat No. DP441, Tiangen Biotech, Beijing, China) and amplified using a ReverTra-Plus-reverse transcription kit (Cat No.FSQ-301, Toyobo, Japan) and backup for post-reverse transcription. Reverse-transcription PCR (qRT-PCR) was used to detect the expression of defense-related genes and the expression of *SPL36* in tissues at each stage, with the *OsActin* gene used as an internal reference (GenBank accession number: NM001058705). The reaction mixtures contained 2 μL cDNA template, 10 μL 2 × SYBR qPCR mix, 0.8 μL each of forward and reverse primers, and ddH_2_O to a final volume of 20 μL. The reaction program was 95 °C for 30 s; 95 °C for 5 s, 55 °C for 10 s; and 72 °C for 5 s for 40 cycles. Each reaction was performed in triplicate, and the relative expression levels of premature senescence-associated genes were calculated using the 2 ^-ΔΔCt^ method. RT-PCR was performed using a quantitative fluorescence gene amplification instrument (qTOWER3G; Jena, Germany). Data were analyzed using SPSS 19.0 software and Excel. Student’s t-test was used to analyze the significance of differences. The primers used for qRT-PCR are shown in Supplementary Table 5.

### Salt Stress Assay

Plate test: Seeds were collected from wild-type Yundao and *spl36* plants, washed, spread on 200 mM NaCl MS medium, and cultured at 28 °C in the light; MS medium without NaCl was used as a control. The assay was performed in triplicate, and the germination rates of the seeds were determined at each stage. After nine days of culture, root lengths were measured and photographed.

Salt stress assay at the seedling stage: Wild-type and mutant seedlings were grown hydroponically for approximately two weeks, and seedlings with roughly the same level of growth were selected for the assay. Wild-type and mutant seedlings were transferred to standard nutrient solution with or without 150 mM NaCl and cultured for four days. The salt-stressed seedlings were transferred to standard nutrient solution and allowed to recover for three days, followed by the detection of plant survival rates, fresh weight, conductivity, and proline content.

## Results

### Phenotype of the Lesion Mimic Mutant *spl36*

**Fig. 1:**
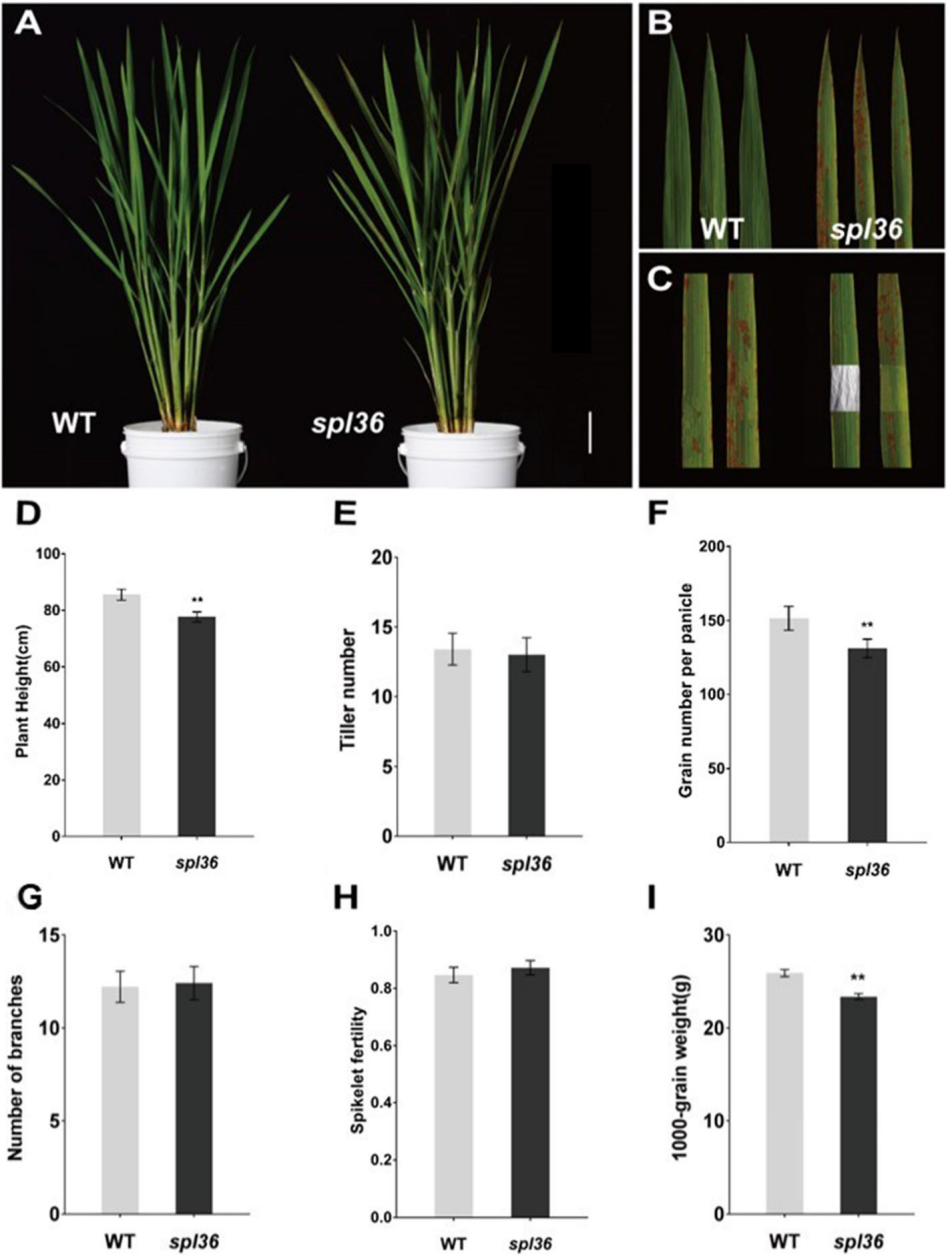
**Phenotypes of the** ***spl36*** **mutant. a** Lesions appear at the tillering stage. Bar = 6 cm. **b** Lesions first appear at the tip of the leaf (WT and *spl36* at the tillering stage). **c** Effect of light on lesion formation under natural conditions; *spl36* before shading (1,2). *spl36* shaded for 7 days (3,4). **d-i** Statistical analysis of important agronomic traits in WT and *spl36* at the maturity stage. Values are means ±SD (*n* = 10); ** indicates significance at *P* ≤ 0.01 by Student’s *t* test

Under normal growing conditions in the summer, *spl36* leaves were not significantly different from those of the wild type before the tillering stage. At the tillering stage, the lesion mimic phenotype appeared in the leaf apex (Fig. 1a). From the tillering stage to the heading stage, these necrotic spots became more severe and gradually spread throughout the leaf (Fig. 1b). To investigate whether lesion formation is induced by light in *spl36*, as in most lesion mimics, we covered *spl36* leaves with 2–3 cm aluminum foil at the tillering stage and used uncovered mutant leaves as controls. After seven days, no spread of lesion mimics had occurred in the covered areas of leaves, whereas lesion mimics appeared on uncovered control leaves (Fig. 1c). These results indicate that the lesion mimic phenotype in *spl36* is induced by light. In addition, the major agronomic traits, including plant height, grain number per panicle, and 1000-grain weight, were significantly reduced in *spl36* vs. wild-type plants (Fig. 1d–i).

### *SPL36* Regulates Plant Growth and Development

**Fig. 2:**
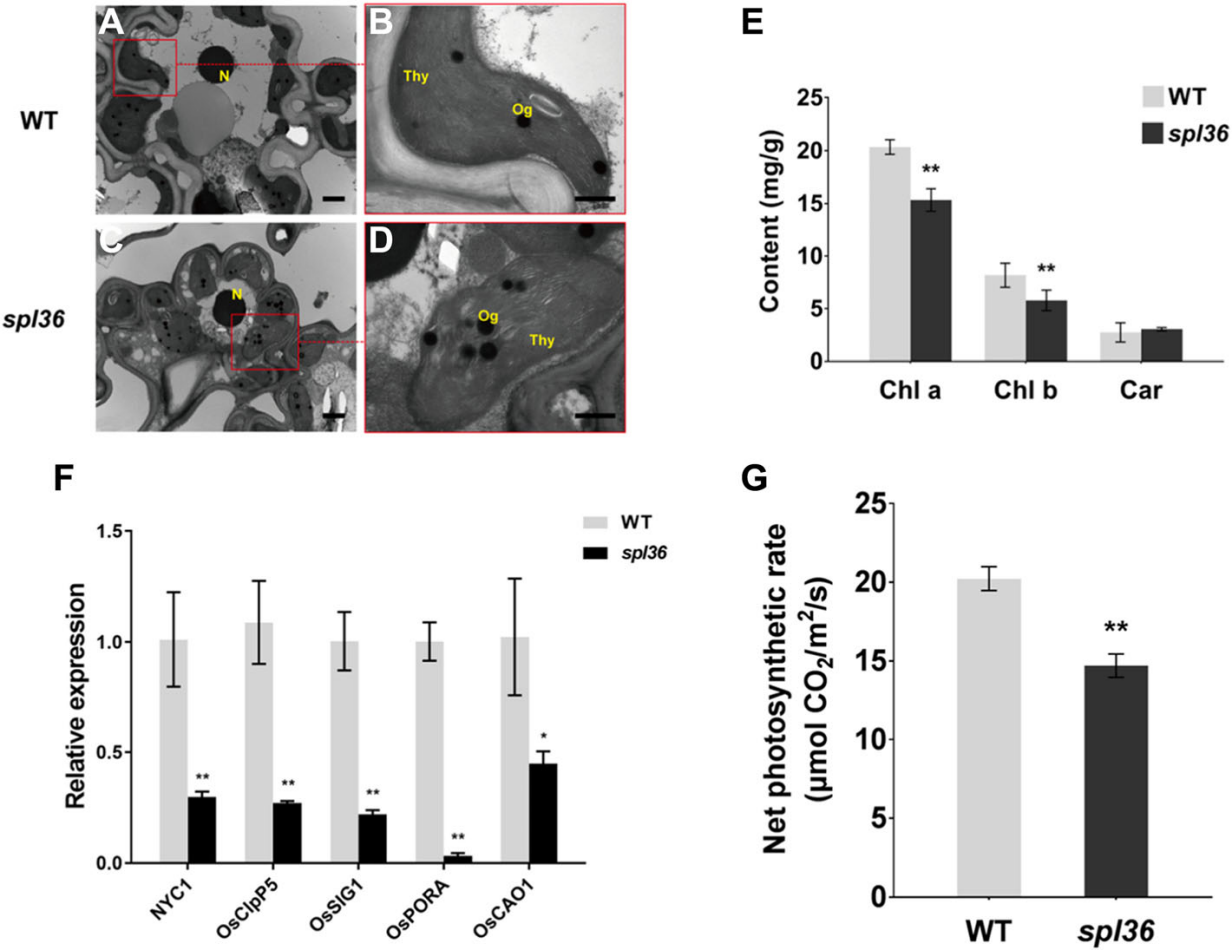
**Chloroplast development and net photosynthetic rate in wild-type and mutant plants. a-d** Chloroplast ultrastructure in wild-type and mutant plants, **a, c**: leaf cells at 6000X; **b, d**: leaf cells at 40,000X; N: nucleus; Thy: chloroplast; Og: osmium granules; Bar = 1 μm. **e** Chlorophyll content in the leaves of wild-type and mutant plants at the tillering stage. **f** Relative expression of chloroplast development and pigment synthesis-related genes. **g** Net photosynthetic rate in wild-type and mutant plants at the tillering stage

Due to the negative agronomic changes in *spl36*, we reasoned that the growth and development of the mutant were affected after the appearance of the lesion mimics because of reduced photosynthesis (Han et al., 2015). We observed chloroplast ultrastructure by transmission electron microscopy and found that *spl36* chloroplasts were atrophied and smaller than those in the WT and had disorganized lamellae (Fig. 2a–d). The contents of both chlorophyll *a* and chlorophyll *b* at the tillering stage were significantly reduced in *spl36* compared to the wild type (Fig. 2e). In addition, the net photosynthetic rates were significantly reduced in *spl36* vs. the wild type (Fig. 2g).

To further explore the effects of the mutation on chloroplast development and chlorophyll biosynthesis, we examined the expression of chloroplast development- and pigment synthesis-related genes in wild-type and mutant plants at the mature stage by qRT-PCR. The expression levels of *NYC1*, *OsClpP5*, *OsSIG1*, *OsPORA*, and *OsCAO1* were significantly reduced in the mutant vs. the wild type (Fig. 2f). These results suggest that *SPL36* influences plant growth and development via its effects on chloroplast structure.

### *SPL36* Regulates ROS Accumulation and Cell Death in Rice

**Fig. 3:**
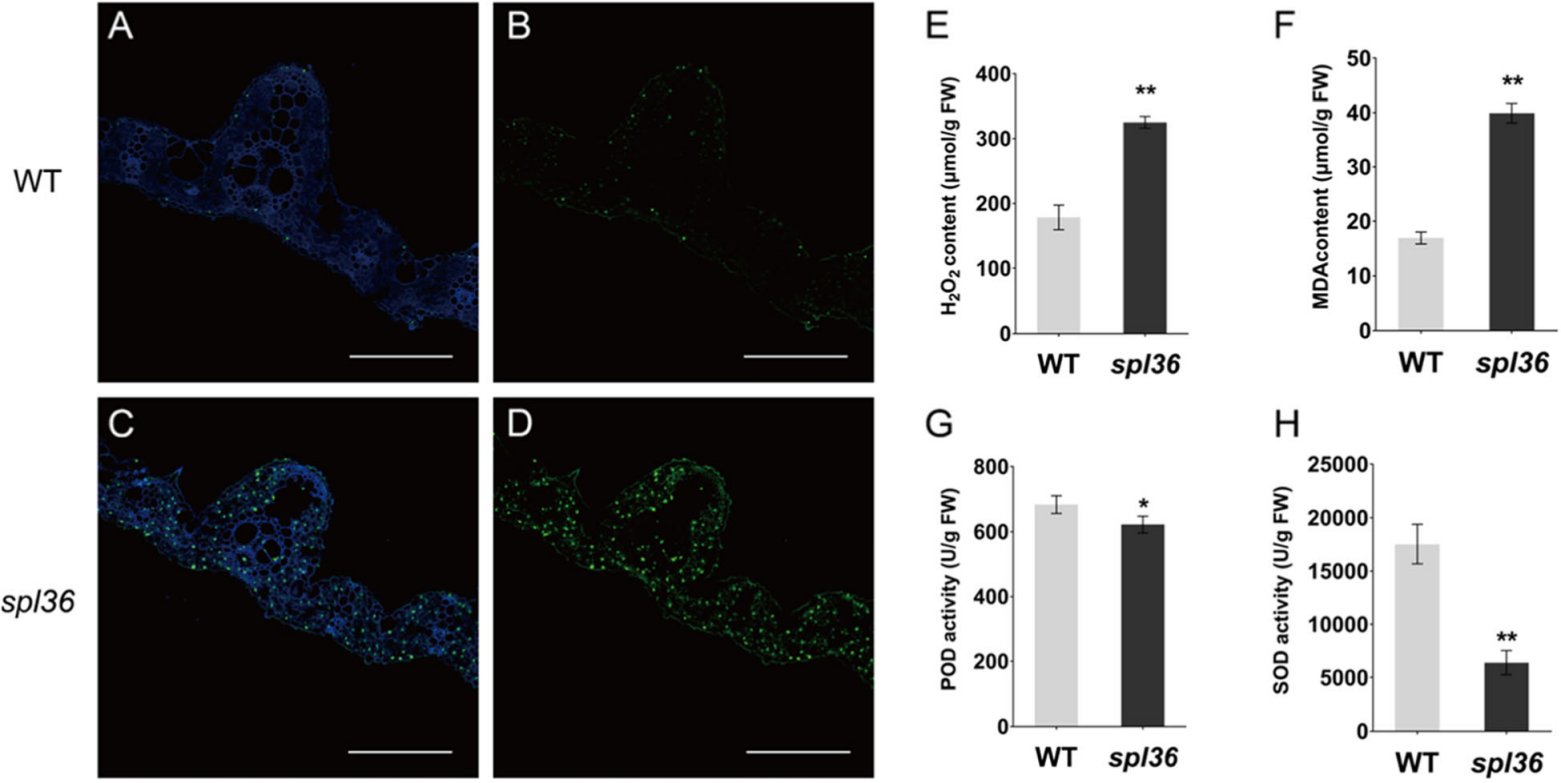
**Physiological and biochemical analysis of wild-type and mutant plants. a- d** TUNEL assay of DNA fragmentation in mesophyll cells. Bar = 100 μm. **e-f** H_2_O_2_ and MDA contents of leaves in *spl36* and WT plants at the heading stage. **g- h** POD and SOD activities in the leaves *spl36* and WT plants at the heading stage. POD: peroxidase; SOD: superoxide dismutase; MDA: malondialdehyde; WT: wild type

The TUNEL (terminal deoxynucleotidyl transferase dUTP nick end labeling) assay is used to detect DNA fragmentation, a marker of programmed cell death (Kim et al., 2009). The TUNEL signal in the nuclei of mutant *spl36* cells was intense and randomly distributed, whereas only a weak TUNEL signal was detected in the wild type (Fig. 3a-d). In addition, the accumulation of reactive oxygen species (ROS) at high concentrations leads to an oxidative burst, which causes cell damage and even triggers programmed cell death (Kim and Coulombe, 2010). H_2_O_2_ contents and peroxidase (POD) activity are directly related to the accumulation of ROS. Superoxide dismutase (SOD) plays an important role in scavenging O^2−^ in plants. We therefore measured H_2_O_2_ content, POD activity, and SOD activity in the plants and found that a large amount of H_2_O_2_ accumulated in *spl36* (Fig. 3e), while POD and SOD activities were significantly reduced in this mutant vs. the wild type (Fig. 3g-h). The reduced activity of these enzymes negatively affects the removal of peroxide and negative oxygen ions, resulting in the accumulation of ROS. In addition, membrane lipid peroxidation occurs when plant organs age or suffer damage under stress. Malondialdehyde (MDA) is the final decomposition product of membrane lipid peroxidation, and therefore MDA content can reflect the degree of damage in stressed plants. The MDA content was significantly higher in *spl36* than in the wild type (Fig. 3f). These results indicate that the lesion mimics in *spl36* mutants are caused by ROS accumulation and irreversible membrane damage.

### *SPL36* Regulates Defense Responses in Rice

**Fig. 4:**
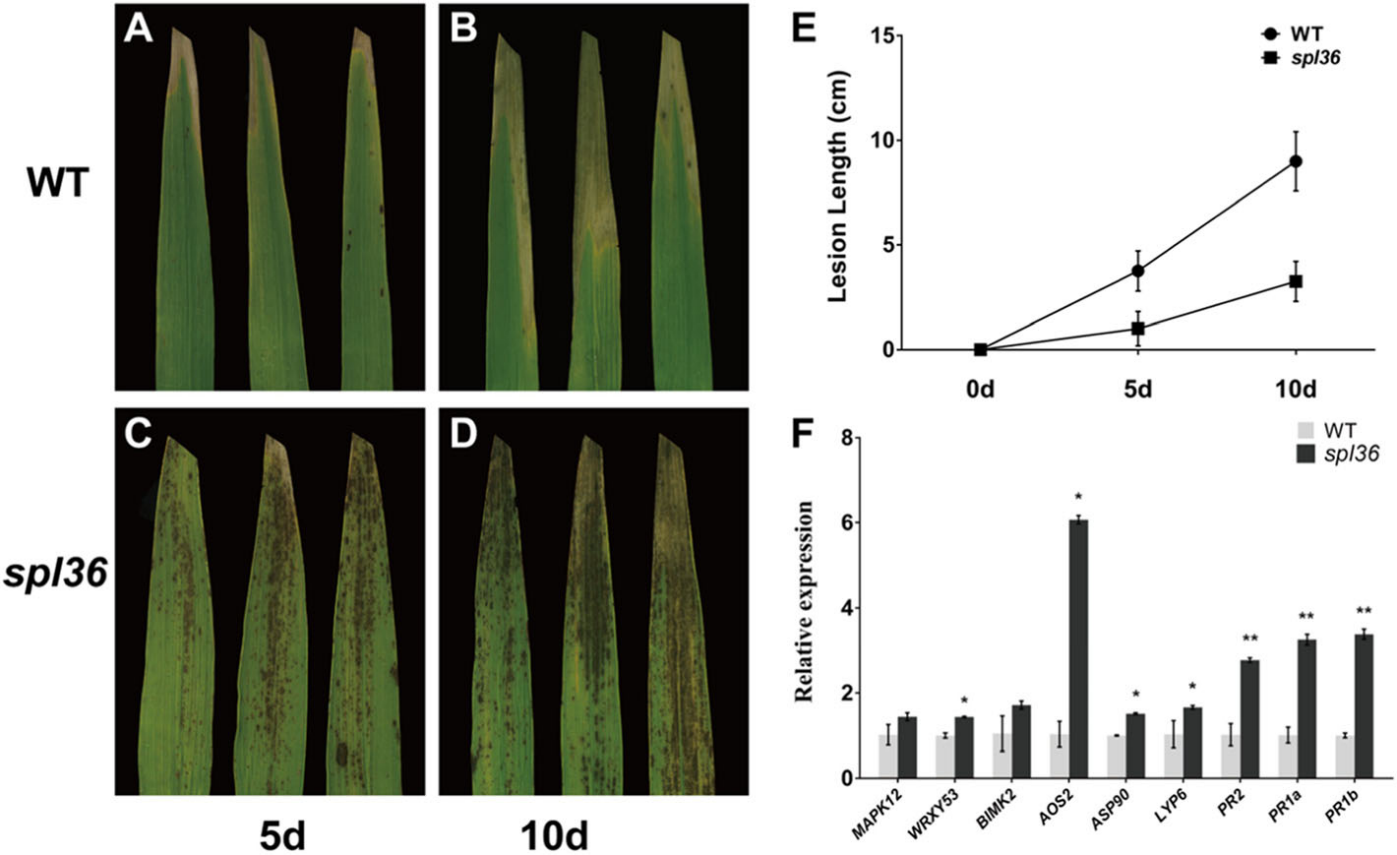
***SPL36*** **regulates defense responses in rice. a-d** Phenotypes of wild-type and *spl36* leaves at 5 days and 10 days after inoculation with the bacterial blight pathogen HM73. **e** Statistical analysis of the length of bacterial leaf blight lesions. **f** Relative expression of defense-related genes in uninoculated plants

Most rice lesion mimic mutants show enhanced resistance to pathogens. To investigate whether *spl36* plants showed increased resistance to rice pathogens, we performed an inoculation assay on wild-type Yundao and *spl36* plants at the tillering stage and used the leaf clipping method to inoculate the plants with rice bacterial blight strain HM73. We detected changes in the inoculation site and length of the lesion mimics in the mutant at 5 and 10 days after inoculation, respectively. At five days after inoculation, the leaf apex of the wild type showed obvious necrotic spots, whereas the mutant did not show obvious disease spots. At 10 days after inoculation, wild-type disease spots were significantly longer than those of the mutant (Fig. 4a-e). These results indicate that resistance to *Xanthomonas oryzae* pv*. Oryzae, Xoo* is significantly enhanced after the emergence of disease spots in *spl36*.

To explore the mechanism underlying the enhanced resistance of *spl36* to bacterial pathogens, we examined the expression of defense-related genes in the wild type and mutants at the tillering stage by qRT-PCR. The expression levels of defense genes *MAPK12*, *WRKY53*, *BIMK2*, *AOS2*, *ASP90*, *LYP6*, *PR2*, *PR1a*, and *PR1b* were significantly elevated in the mutants (Fig. 4f). Thus, the loss of function of the *SPL36*-encoded protein triggers a defense response in rice, leading to the enhanced resistance of *spl36* to pathogens.

### Genetic Analysis and Map-Based Cloning of *SPL36*

**Fig. 5:**
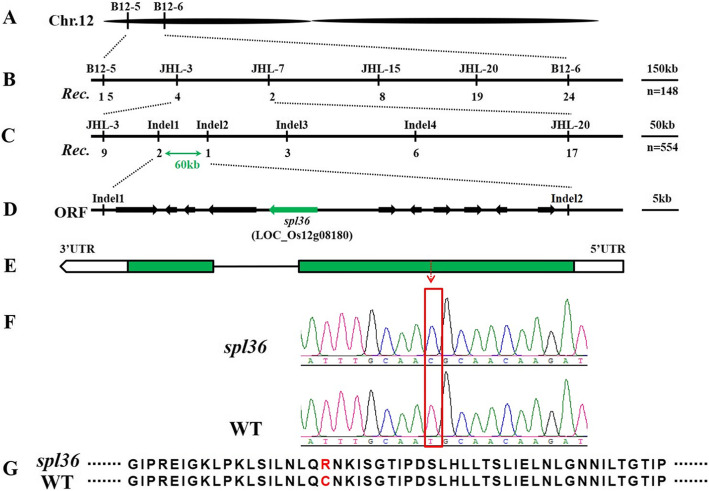
**Genetic and physical maps of the** ***SPL36*** **gene. a** The *SPL36* gene was localized to chromosome 12 between InDel markers B12–5 and B12–6. **b** The *SPL36* gene was delimited to the JHL-3 to JHL-7 interval on chromosome 12 using 148 F_2_ mutant individuals; marker names and number of recombinants (*Rec.*) are shown. **C** Fine genetic mapping of the *SPL36* gene based on 554 mutant F_2_ individuals. **d** 11 putative ORFs located in the ~ 60-kb region of this locus. **e** Gene structure of *LOC_Os12g08180*. **f** Sequence analysis of the T-to-C mutation site in wild type and *spl36*. **g** The mutation results in a change from cysteine to arginine in the encoded protein

We hybridized *spl36* as the female parent with ZF802 of *japonica* cultivar TN1. The F_1_ plants did not show lesion mimics. The segregation ratio of the normal phenotype to lesion mimic phenotype in the F_2_ population was essentially in compliance with a 3:1 ratio, indicating that the *spl36* phenotype is caused by a mutation in a single recessive nuclear gene (Supplementary Table 1). We used a selection of polymorphic markers from 238 insertion and deletion tags to map the mutant gene in 21 F_2_ individuals with a lesion mimic phenotype and mapped the mutation site to a location between B12–5 and B12–6 on chromosome 12 (Fig. 5a). The *SPL36* location was further refined to a region between JHL-3 and JHL-7 by genotyping 148 mutant F2 individuals from the same cross and adding four additional polymorphic tags (Fig. 5b). Using 554 additional F_2_ mutant individuals and four newly developed polymorphic tags, we ultimately mapped *SPL36* to a 60 kb region between markers InDel1 and InDel2 (Fig. 5c). Analysis using https://rice.plantbiology.msu.edu/ predicted that this region contains 11 open reading frames (ORFs) encoding seven expressed proteins and four functional proteins (Fig. 5d). Sequencing and alignment revealed that in *spl36*, gene *LOC_Os12g08180* was mutated (Fig. 5e): nucleotide T at position 1462 in the coding region of this gene was replaced by C (Fig. 5f), resulting in the change of the encoded amino acid from cysteine to arginine (Fig. 5g). Therefore, *LOC_Os12g08180* is the candidate gene for *SPL36*.

### Functional Complementation of the *spl36* Mutant with *LOC_Os12g08180*

**Fig. 6:**
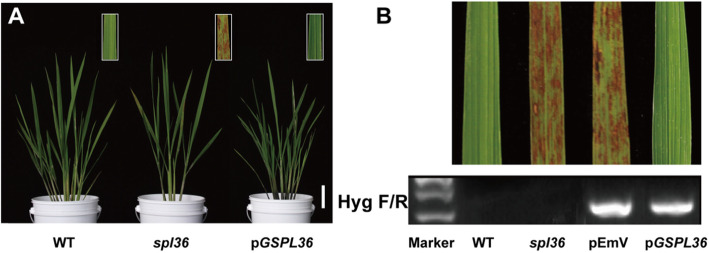
**Functional complementation of the** ***spl36*** **mutant with** ***LOC_Os12g08180.*** **a** The phenotype of the *spl36* mutant transformed with the genomic sequence of *SPL36* (*pGSPL36*) was completely recovered to that of the wild type. The insets show enlarged views of leaf sections with lesion spots. Bar = 8 cm. **b** Transgenic plants were verified by the presence of the hygromycin selectable marker gene. pEmV: empty vector

To determine whether the single base substitution in *LOC_Os12g08180* is indeed associated with the *spl36* phenotype, we constructed the vector *pGSPL36*, which contained genomic DNA fragments including the promoter of the *SPL36* gene in wild-type Yundao, and introduced it into *spl36* by *Agrobacterium tumefaciens*-mediated transformation. The corresponding empty vector pEmV was used as a control. Of the 60 T0 plants, 54 were positive transformants. All of these transformants had the same phenotype as the wild type (Fig. 6a), while plants transformed with the control vector showed the same lesion mimic phenotype as *spl36* (Fig. 6b). These results demonstrate that *LOC_Os12g08180* is *SPL36* and that the single base substitution in *spl36* leads to the appearance of the lesion mimic phenotype of the mutant.

### Expression Analysis of *SPL36*

**Fig. 7:**
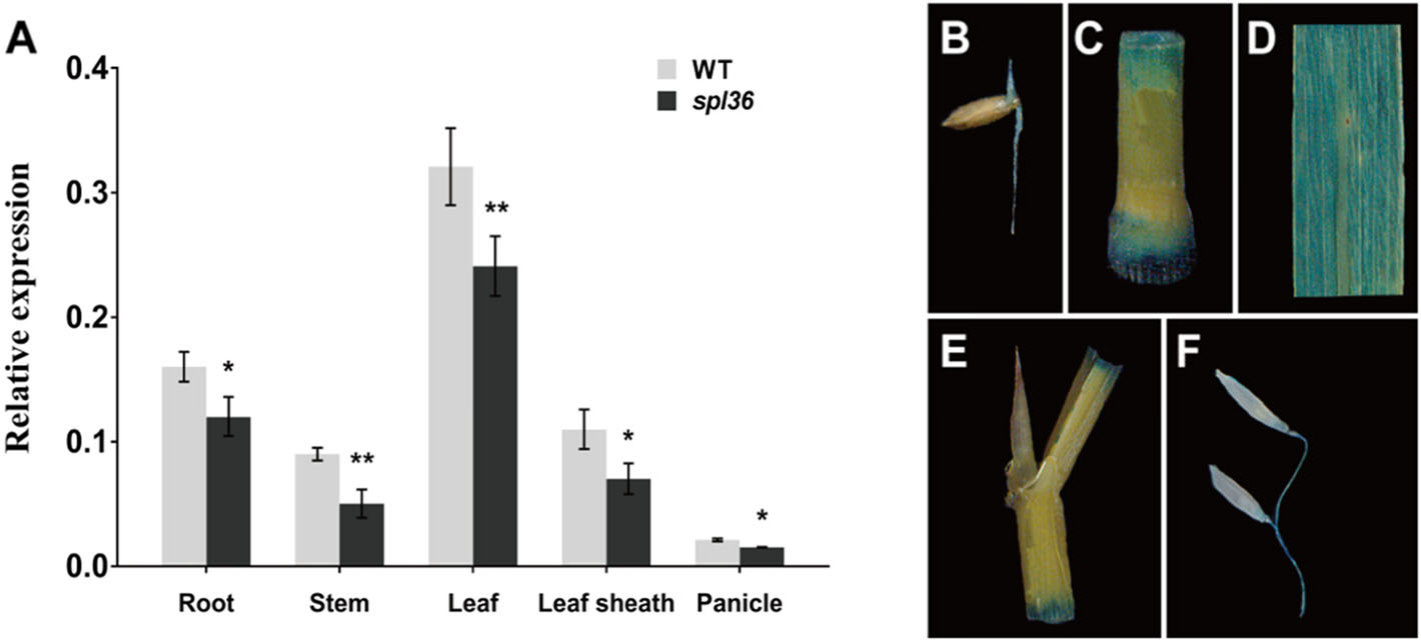
**Expression analysis of** ***SPL36.*** **a** Expression of *SPL36* in various organs of wild type and *spl36* plants analyzed by quantitative RT-PCR. **b-f** Histochemical signals from the *SPL36* promoter-GUS reporter gene. GUS signals were detected in the root **b**, stem **c**, leaf **d**, leaf sheath **e**, and panicle **f**

We performed reverse-transcription quantitative PCR (qRT-PCR) to analyze the expression of *SPL36* in various organs. *SPL36* was expressed in all organs, with higher expression levels in leaves, leaf sheaths, and roots and lower expression levels in stems and panicles. *SPL36* was expressed at significantly higher levels in all organs of *spl36* compared to wild-type Yundao (Fig. 7a). To analyze the spatiotemporal expression pattern of *SPL36* more precisely, we constructed the vector p*SPL36*::GUS by fusing the GUS gene with the promoter of *SPL36* from the wild type. We then performed *Agrobacterium tumefaciens*-mediated transformation to obtain transgenic plants. We stained various organs of the transgene-positive plants and observed GUS signal in various tissues (Fig. 7b-f). Our results showed that *SPL36* was expressed in tested organs and tissues including the root, leaf, leaf sheath, stem and panicle. Notably, *SPL36* transcript level was more abundant in the leaf relative to other tissues or organs. Relative strong GUS signals were observed in the leaf, whereas weak signals were also found in the root, culm, leaf sheath, and panicle. These findings were consistent with the RT-qPCR results.

### Subcellular Localization of *SPL36*

**Fig. 8:**
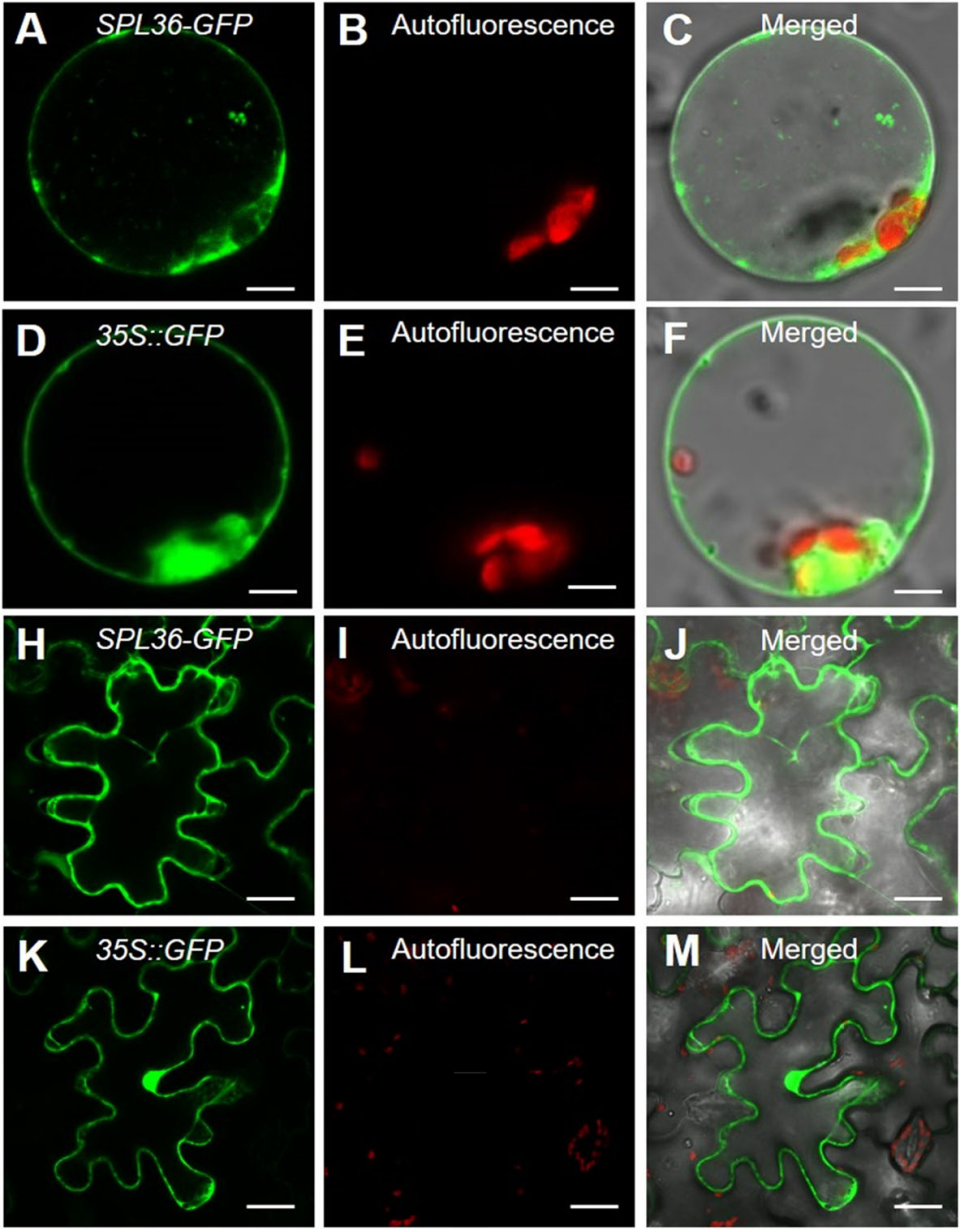
**Subcellular localization of SPL36. a-c** Transient assay in rice protoplasts. **h-j** *N. benthamiana* leaf assay. **d-f** and **k-m** *35S::GFP* controls used in the assays. *SPL36-GFP*: *SPL36* GFP fusion protein. Bar = 20 μm

To determine the subcellular location of *SPL36*, we fused the full-length coding sequence of *SPL36* to the N-terminus of green fluorescent protein (GFP). When transiently expressed in rice protoplasts, the GFP signal appeared on the plasma membrane and cytoplasm and the signal of *35S*::*GFP* appeared on membrane, cytoplasm, and nucleus (Fig. 8a–d). To verify this observation, we transformed *Nicotiana benthamiana* leaves with a plasmid containing the *SPL36*-*GFP* fusion vector. *SPL36*-*GFP* protein was detected on the membrane and cytoplasm and *35S*::*GFP* protein was detected on the membrane, cytoplasm, and nucleus (Fig. 8e–h). These results indicate that *SPL36* localizes to the membrane and cytoplasm.

### *SPL36* Is Involved in Salt Stress Responses in Rice

After verifying that the single base substitution of *LOC_Os12g08180* was responsible for the lesion mimic phenotype of *spl36*, we determined that this gene encodes a receptor-like protein kinase. Plant receptor-like protein kinases play regulatory roles in plant growth and development and disease resistance (Afzal et al., 2008; Li Liyun et al., 2008), and most of them function in stress responses. To investigate whether *SPL36* is involved in stress response-related pathways, we performed salt stress assays in which we placed wild-type and mutant seeds in flat dishes for the germination assay and grew seedlings hydroponically in the absence and presence of NaCl for the seedling assay.

**Fig. 9:**
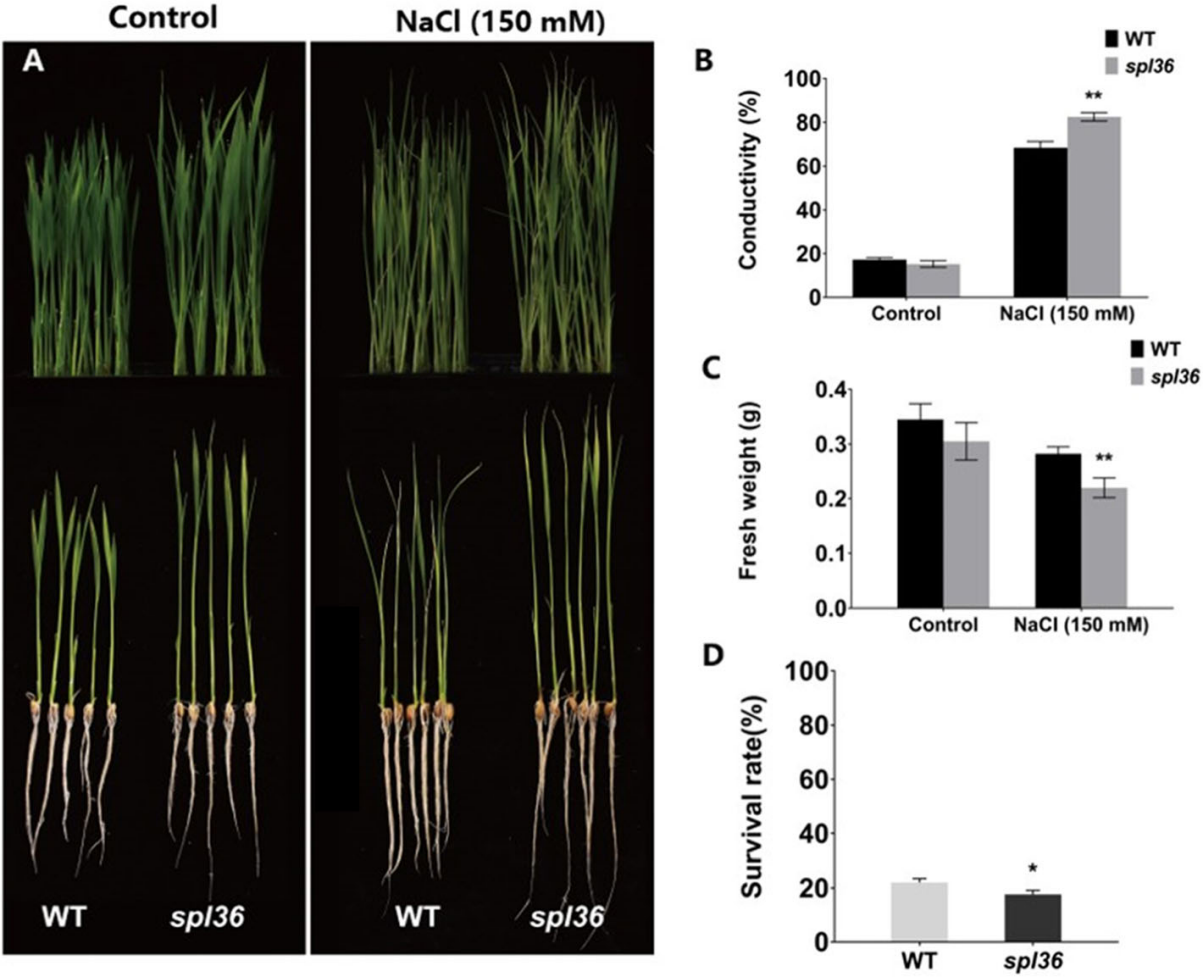
Analysis of salt-stress responses in wild type and spl36 plants at the seedling stage. **a** Phenotypes wild-type and mutant seedlings before and after 150 mM NaCl treatment. **b** Conductivity of wild-type and mutant seedlings before and after 150 mM NaCl treatment. **c** Fresh weights of wild-type and mutant seedlings before and after 150 mM NaCl treatment. **d** Survival rates of wild-type and mutant seedlings after 150 mM NaCl treatment

In the absence of salt treatment, no significant difference in the germination rates of *spl36* vs. wild-type seeds was detected over a one-week period. Under salt treatment, the germination rates of both mutant and wild-type seeds decreased significantly, while the germination rate of the wild type was also significantly lower than that of the mutant. After 9 days of culture in the germination assay, we measured the lengths of the root portions of salt-treated and control seedlings. Under control conditions, there was no significant difference in stem length between wild-type and mutant seedlings. By contrast, under NaCl treatment, the stems were significantly shorter in mutant vs. wild-type seedlings (Supplementary Fig. 1). We also grew wild-type and mutant seedlings hydroponically for four weeks, cultured them in high-salt medium for four days, returned them to normal conditions for recovery, and examined them three days later. No significant changes were detected in the wild type following four days of treatment, with the phenotype recovering after the return to normal conditions. By contrast, while *spl36* showed significant leaf bending after salt treatment, and the plants did not recover or even died following their return to normal conditions. Our analysis of fresh weight, conductivity, and final survival of plants before and after treatment and under control conditions revealed that *spl36* was more sensitive to salt treatment than the wild type (Fig. 9). In summary, *SPL36* is involved in salt-stress responses in rice.

## Discussion

Lesion mimic mutants are extremely valuable for studying PCD and defense-related responses in plant cells. In the present study, we selected the lesion mimic mutant *spl36* from a mutant library generated by mutagenizing wild-type Yundao rice using EMS. There were no obvious phenotypic differences between this mutant and the wild type at the seedling stage. However, reddish-brown disease spots appeared in the mutant at the leaf apex at the tillering stage and gradually spread throughout the leaf. We observed the chloroplast ultrastructures of both the wild type and mutant at this stage and measured their photosynthetic rates. The appearance of lesion mimics led to significant changes in chloroplast structure in the mutant. Since chloroplasts are the sites of photosynthesis (Wu et al., 2018), the appearance of lesion mimics affected both the growth and development of the plants. Altered chloroplast structure is also a direct cause of the decline in multiple agronomic traits in plants (Ishikawa et al., 2001).

Using map-based cloning, we mapped the *SPL36* gene within a 60 Kb interval on chromosome 12. Based on information in the rice genome database (https://rice.plantbiology.msu.edu/), this interval contains 11 ORFs, including genes for seven expressed proteins and four functional proteins. We amplified the genomic sequences in this region in the mutant and wild type by PCR. Sequence alignment and sequencing analysis revealed that nucleotide T at position 1462 in the coding region of the gene *LOC_Os12g08180* was replaced with C, resulting in a change in the encoded amino acid from cysteine to arginine. Using a functional complementation assay, we determined that this gene is *SPL36*. Structural analysis of the protein encoded by this gene showed that this protein is a receptor-like protein kinase receptor containing multiple leucine-repeat domains (Supplementary Fig. 2).

Leucine-rich receptor-like kinases (LRR-RLKs) are closely related to stress and defense responses in plants. The *PRK1* gene was initially isolated from Arabidopsis in 1997 by Hong et al. (Hong et al., 1997), who demonstrated that the LRR domain of *PRK1* functions in protein-protein interactions, as well as the perception of stress signals from the environment. In 2014, Yang et al. (Yang et al., 2014) identified new LRR-RLKs in wild soybean and demonstrated that *GsLRPK* improves drought resistance when exogenously expressed in Arabidopsis. *OsGIRL1* is upregulated in response to abiotic stresses including salt, osmotic stress, and heat, and the stress-related hormones salicylic acid and abscisic acid but is downregulated in response to jasmonic acid; *OsGIRL1* is located in the plasma membrane. The biological function of *OsGIRL1* was explored by exogenously expressing this gene in Arabidopsis plants and examining plant responses to irradiation, salt pressure, osmotic pressure, and thermal stress (Park et al., 2014). The LRR type receptor protein kinase gene *OsRLK1* is induced by low temperature and salt stress in rice (Lee et al., 2004). *CALRR1* in pepper is induced not only by exposure to anthrax pathogens but also under abiotic stress conditions such as high salt, abscisic acid, and wounding (Jung et al., 2004). Here, using two different salt stress protocols, we demonstrated that *spl36* was more sensitive than the wild type to salt treatment. Although these results can be explained by the finding that a missense mutation in the coding region of *LOC_Os12g08180* led to the loss of protein function, the specific mechanism remains to be elucidated and will be explored in the future.

LRR-RLKs are primarily associated with abiotic stress responses in plants, while their relationships with PCD and disease resistance have not been reported. We verified the higher frequency of cell death in *spl36* by performing a TUNEL assay and measured H_2_O_2_ and MDA levels and POD and SOD activities in the wild type and mutant. *spl36* accumulated more ROS than the wild type, which led to an oxidative burst and ultimately PCD. Since lesion mimics arise spontaneously in *spl36*, our findings indicate that *SPL36* negatively regulates PCD in rice.

Most previously reported lesion mimic mutants show some enhancement of disease resistance. To investigate whether *SPL36* is involved in disease resistance in rice, we used the leaf clipping method to inoculate wild-type and mutant plants with the bacterial blight pathogen HM73 and found that *spl36* had significant resistance to this pathogen. However, it remains to be determined whether *spl36* has broad-spectrum resistance. We also analyzed the expression of several defense-related genes in the wild type and mutant. The defense genes *MAPK12*, *WRKY53*, *BIMK2*, *AOS2*, *LYP6*, *PR1a*, and *PR1b* were significantly upregulated in the mutant vs. the wild type. *OsWRKY53* encodes a transcriptional activator that plays an important role in the excitation-induced defense signal transduction pathway in rice (Chujo et al., 2007; Tian et al., 2017). *OsAOS2* expression in leaves is significantly induced by rice blast. The expression of *OsAOS2* driven by the *PBZ1* promoter activated the expression of other pathogenesis-related genes, thereby increasing resistance to rice blast (Mei et al., 2006). *OsBIMK2* plays an important role in rice disease resistance responses (Song et al., 2006). *LYP6* encodes a protein containing cytolytic enzyme motifs that functions as a pattern recognition receptor for bacterial peptidoglycan and fungal chitin and plays a dual role in the recognition of peptidoglycan and chitin during innate immunity in rice (Liu et al., 2012). *OsPR1a* and *OsPR1b* are pathogenesis-related genes (Agrawal et al., 2000). Based on our results, we propose that *SPL36* regulates the disease resistance response in rice by upregulating the expression of defense genes, but the specific mechanism requires further investigation.

## Conclusion

We cloned a novel spotted leaf gene (*SPL36*) encoding a receptor-like protein kinase that contains repeated leucine domains and may be involved in stress responses in rice. This is the first report of the involvement of a receptor-like protein kinase in disease resistance-related pathways in rice. We demonstrated that the loss of function of *SPL36* results in enhanced resistance to pathogens and increased salt sensitivity. We are currently conducting an in-depth study to determine whether the *spl36* mutant has broad-spectrum resistance to pathogens and to uncover the role of *SPL36* in the salt stress response in rice.

## Supplementary Information

**Additional file 1: Table S1.** Genetic analysis of the lesion mimic phenotype in F_2_ populations. **Table S2.** Distribution of primers used to detect polymorphisms on each chromosome. **Table S3.** Primers used for mapping. **Table S4.** Primers used for vector construction. **Table S5.** Primers used for qRT-PCR. **Figure S1.** Analysis of salt stress in wild type and *spl36***. A** Wild-type and mutant seeds on 200 mM NaCl at 9 days of culture. **B** Analysis of relative germination rates of wild-type and mutant seeds after salt stress. **C** Analysis of growth potential in wild type and mutant seeds on 200 mM NaCl at 9 days of culture. **D** Analysis of stem length in wild type and mutant plants after 9 days of salt stress treatment. **Figure S2.** Structural prediction of the protein encoded by *SPL36***. A** Predicted protein structure of SPL36. **B** Protein domains of SPL36. **C** Alignment of the conserved amino acid sequences of SPL36 homologs in various organisms.

## Data Availability

All data generated or analyzed during this study are included in this published article and its additional files.

## Abbreviations

EMS: Ethyl methyl sulfonate
GFP: Green fluorescent protein
GUS: β-glucuronidase
LRR-RLK: Leucine-rich repeat receptor-like protein kinase
PCR: Polymerase chain reaction
PCD: Programmed cell death
POD: Peroxidase
qRT-PCR: Quantitative reverse-transcription polymerase chain reaction
QTL: Quantitative trait locus
RNA: Ribonucleic acid
ROS: Reactive oxygen species
SDS: Sodium dodecyl sulfate
SOD: Superoxide dismutase
TUNEL: Terminal-deoxynucleotidyl transferase dUTP nick end labeling
