# Supplementary material for: SPL36 Encodes a Receptor-like Protein Kinase that Regulates Programmed Cell Death and Defense Responses in Rice
Source: Rice (N Y). 2021 Apr 7;14:34. doi: 10.1186/s12284-021-00475-y (PMC8026784; doi:10.1186/s12284-021-00475-y)
Supplement: Supplementary file 1 — Additional file 1: Table S1. Genetic analysis of the lesion mimic phenotype in F2 populations. Table S2. Distribution of primers used to detect polymorphisms on each chromosome. Table S3. Primers used for mapping. Table S4. Primers used for vector construction. Table S5. Primers used for qRT-PCR. Figure S1. Analysis of salt stress in wild type and spl36. A Wild-type and mutant seeds on 200 mM NaCl at 9 days of culture. B Analysis of relative germination rates of wild-type and mutant seeds after salt stress. C Analysis of growth potential in wild type and mutant seeds on 200 mM NaCl at 9 days of culture. D Analysis of stem length in wild type and mutant plants after 9 days of salt stress treatment. Figure S2. Structural prediction of the protein encoded by SPL36. A Predicted protein structure of SPL36. B Protein domains of SPL36. C Alignment of the conserved amino acid sequences of SPL36 homologs in various organisms. [file 12284_2021_475_MOESM1_ESM.docx]

Supplementary Table 1

Genetic analysis of the lesion mimic phenotype in F_2_ populations

| Cross | No. of F_2_ individuals | | | χ^2^_0.05_<3.841 |
| --- | --- | --- | --- | --- |
|  | Normal | Lesion mimics | Total |  |
| *spl36*×TN1 | 721 | 229 | 950 | 0.406 |
| *spl36*×ZF802 | 830 | 282 | 1112 | 0.076 |

Supplementary Table 2

| Cross | Chromosome | | | | | | | | | | | | |
| --- | --- | --- | --- | --- | --- | --- | --- | --- | --- | --- | --- | --- | --- |
|  | 1 | 2 | 3 | 4 | 5 | 6 | 7 | 8 | 9 | 10 | 11 | 12 | Total |
| *spl36*×TN1 | 19 | 11 | 16 | 10 | 8 | 14 | 10 | 13 | 9 | 11 | 7 | 8 | 136 |

Distribution of primers used to detect polymorphisms on each chromosome

Supplementary Table 3 Primers used for mapping

| Primer name | Forward primer (5′→3′) | Reverse primer (5′→3′) |
| --- | --- | --- |
| B12-5 | ATGAAAGTCGGTGACGATGG | ACTTAGGGGATCAGGGGATG |
| B12-6 | AGCGTGTGACTTCATTGCAC | GGTGATCACCAGCAACACAC |
| JHL-3 | GAGACGACGGTAGCGGAC | CATCGCCATACACCGCTG |
| JHL-7 | TCTATCGATCTGTGTAAGGAGCT | GCAATTACATAAATGCGGCTAGG |
| JHL-15 | AGACAGAAACAAATTGATAGAG | TGGATTGTTGCATTAGTTCAGTT |
| JHL-20 | TTCAGATTGATGTCATTTAAACCAT | TCGAAACTAGGGAGCTGTTCA |
| Indel1 | AAGTTGGCATTTTATATGTA | CTGTGTTGTTCTGTCACTCT |
| Indel2 | CTCTCATCCTAAAATACAA | AAACCCATTTTTATTAAGAT |
| Indel3 | ATCAGCAGGTAGCAGCAATG | AACGAACAAGGGTAGTACTGTGC |
| Indel4 | CCCCCATTATGTTTGCAGTT | TGTGACCATCAGAGCAAAGG |

Supplementary Table 4 Primers used for vector construction

| Primer name | Primer sequence (5′→3′) |
| --- | --- |
| SPL36-CPT- EcoRI-F | CATGATTACGAATTCACGAGTGAGAGGCAAATGGA |
| SPL36-CPT- HindIII-R | GCCAGTGCCAAGCTTTCCTGCATGCCTGTGAGTAA |
| SPL36-GFP-KpnI-F | TTCGAGCTCGGTACCATGGGAGGCCAAGAACAGGT |
| SPL36-GFP-BamHI-R | CTTGCTCACGGATCCTCACTTTGCGTTGAATAGCA |
| SPL36-GUS- EcoRI-F | CATGATTACGAATTCACGAGTGAGAGGCAAATGGA |
| SPL36-GUS- HindIII-R | GCCAGTGCCAAGCTTGCTTCGGCGTTGGGAGTCCAGT |

Supplementary Table 5 Primers used for qRT-PCR

| Primer name | Forward primer (5′→3′) | Reverse primer (5′→3′) |
| --- | --- | --- |
| *OsActin* | TGGCATCTCTCAGCACATTCC | TGCACAATGGATGGGTCAGA |
| *NYC1* | AACACTGCAAAGCTGGACCT | TCAAGCTCGTTGATGGTCTG |
| *OsClpP5* | TGCTGCATCACAAGGCTAAC | AAGGGCTTTAAGGGGATTCA |
| *OsSIG1* | TTTCTTGCAAGGGAGATGCT | GCCAACTCAACACCAAGGTT |
| *OsPORA* | AGGCGTACAAGGACAGCAAG | TGTTCCAGCTCCAGTACACG |
| *OsCAO1* | TGGCAAAAGCTCACAATCAG | ACAAGGGTCTCCATTTGTGC |
| *MPK12* | TGACCAAGAGAGGAGTGCAG | CGTCATCGTTGTGCACTAGG |
| *WRKY53* | TACTACAAGTGCACGACGGT | GAGCATCTCGAGGTGTAGG |
| *BIMK2* | GGTACCTCAGCCACTTTCAT | TCCATAGCTTCCCTTGCCAA |
| *AOS2* | GAGAGACGGAGAACCCTAGC | GAAGTGATGGCCGGCTTAAG |
| *ASP90* | CCTTCCAACTGAGGTCGAGT | AGACTGCAGGCTGTGTAAGA |
| *LYP6* | AACTGCTGGAAATGTGTGC | TTGAAGACCAGAGGAGAGACG |
| *NPR2* | CGGTACAAGTAGGAGGAGCT | GCTCGACGTTGAACCTGATC |
| *PR1a* | CATCACCTGCAACTACTCGC | TCTCACCAGCATACGTCGT |
| *PR1b* | ATCCCAAGTCCTGCGTACAA | AGACTGCAGGCTGTGTAAGA |

Supplementary Figure 1 Analysis of salt stress in wild type and *spl36*

**A** Wild-type and mutant seeds on 200 mM NaCl at 9 days of culture. **B** Analysis of relative germination rates of wild-type and mutant seeds after salt stress. **C** Analysis of growth potential in wild type and mutant seeds on 200 mM NaCl at 9 days of culture. **D** Analysis of stem length in wild type and mutant plants after 9 days of salt stress treatment.

Supplementary Figure 2 Structural prediction of the protein encoded by *SPL36*

**A** Predicted protein structure of SPL36. **B** Protein domains of SPL36. **C** Alignment of the conserved amino acid sequences of SPL36 homologs in various organisms.
